# Synthesis and Antimicrobial Activity of Sulfur Derivatives of Quinolinium Salts

**DOI:** 10.3390/molecules23010218

**Published:** 2018-01-20

**Authors:** Anna Empel, Ewa Kisiel, Robert D. Wojtyczka, Małgorzata Kępa, Danuta Idzik, Aleksander Sochanik, Tomasz J. Wąsik, Andrzej Zięba

**Affiliations:** 1 Department of Organic Chemistry, School of Pharmacy and Division of Laboratory Medicine in Sosnowiec, Medical University of Silesia in Katowice, Jagiellońska 4, 41-200 Sosnowiec, Poland; anna.nycz00@gmail.com (A.E.); e_kisiel@o2.pl (E.K.); 2 Department and Institute of Microbiology and Virology, School of Pharmacy and Division of Laboratory Medicine in Sosnowiec, Medical University of Silesia in Katowice, Jagiellońska 4, 41-200 Sosnowiec, Poland; rwojtyczka@sum.edu.pl (R.D.W.); mkepa@sum.edu.pl (M.K.); didzik@sum.edu.pl (D.I.); twasik@sum.edu.pl (T.J.W.); 3 Center for Translational Research and Molecular Biology of Cancer, Maria Skłodowska-Curie Mamorial Cancer Center and Institute of Oncology, Wybrzeże AK 15, 44-101 Gliwice, Poland; aleksander.sochanik@io.gliwice.pl

**Keywords:** quinolinium salts, antimicrobial activity, minimal inhibitory concentration

## Abstract

A novel method for cleavage of the dithiine ring in 5,12-(dimethyl)-thioqinantrenium bis-chloride **1** “via” reaction with sodium hydrosulfide leads to 1-methyl-3-mercaptoquinoline-4(1*H*)-thione **2**. Further transformation of thiol and thione functions of compound **2** leads to a series of sulfide and disulfide derivatives of quinolinium salts **4** and **6**. 1-Methyl-4-chloro-3-benzylthioquinoline chloride **8** was obtained by *N*-alkylating 4-chloro-3-benzylthioquinoline using dimethyl sulfate. Antimicrobial activity of the obtained compounds was investigated using six Gram-positive and six Gram-negative bacterial strains, as well as *Candida albicans* yeast. Greater activity was demonstrated towards Gram-positive strains. MIC values for compounds and with benzylthio **4d** and benzoylthio **4f** substituents in 3-quinoline position were found to be in the 0.5–1 μg/mL range, at a level similar to that of ciprofloxacin (reference). Compounds **4d** and **4f** also demonstrated interesting antifungal properties (MIC = 1).

## 1. Introduction

Increasing bacterial resistance to antibiotics has become a very serious medical problem for health care systems worldwide [1,2]. This situation requires continued efforts in searching for novel classes of compounds with antimicrobial activities. The rising number of reports concerning microbial resistance to antibiotics has led to the expression of concerns over imminent incurability of some pathogen-caused diseases. Quinoline stands out prominently among pharmacologically active compounds [3]. Its structural motif is present in many naturally-occurring compounds that show interesting biological properties. Quinine, the first effective antimalarial drug isolated from cinchona tree bark, provides a good example. Based on its established structure novel synthetic antimalarial drugs were obtained, such as chloroquine, primaquine and mefloquine [4]. Other examples of similar compounds include streptonigrin, produced by *Streptomyces flocculus*, an aminoquinone antitumor and antibacterial antibiotic [5] and camptothecin, a cytotoxic quinoline alkaloid isolated from the bark and stem of *Camptotheca acuminata* (Camptotheca, happy tree). Derivatives of the latter, topotecan and irinotecan, are anticancer drugs [6]. An important group of chemotherapeutics with quinoline motif are 4-quinolone derivatives effective against Gram-negative bacteria [7,8]. Quinoline ring systems have often been included when designing novel synthetic or semi-synthetic compounds with different pharmacological properties. Numerous quinoline derivatives, offering a broad spectrum of activity, have been reported. They include compounds with anticancer [9,10,11], antimycobacterial [12,13], antimicrobial [14,15], anticonvulsant [16,17], antiinflamatory [17,18] and cardiovascular [19,20] activity. 

Substantial antimicrobial activity was demonstrated by 4-aminoquinoline salts [21,22,23]. There have been also reports concerning such activity in cases of sulfide derivatives of quinoline [24]. In our previous papers, we described the synthesis of 1,3,4-trisubstituted derivatives of quinoline salts [25,26]. Since initial trials demonstrated their strong antimicrobial properties we decided to investigate and report in here the activity of such derivatives against a wider panel of reference strains; we also report synthesis of novel derivatives. 

## 2. Results and Discussion

### 2.1. Chemistry

**Scheme 1:**
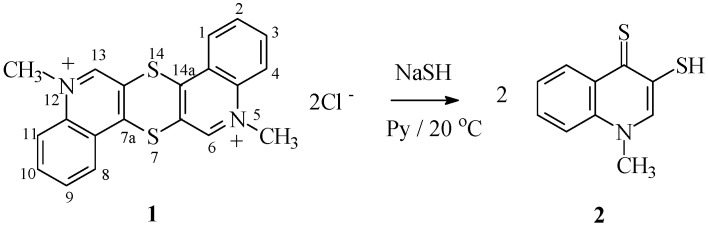
Synthesis of compounds **2**.

5,12-Dialkylthioquintrenediinium bis-salts **1** were obtained by alkylating thioquinantrene (1,4-dithiino[2,3-c,5,6-c′]diquinoline) [24]. The previously described reaction of thioquinantrene ring opening requires gaseous hydrogen sulfide. The novel method described herein based on using sodium hydrosulfide is safer, less cumbersome, and does not require disposition of excess hydrogen sulfide. Reactions of salts **1** with sodium hydrosulfide in pyridine solution at room temperature occur with complete consumption of salts **1**. The reaction occurs by nucleophilic attack of hydrosulfide anion on *aza*-activated positions 7a and 14a of salts **1**, which leads to the opening of the 1,4-dithiin ring of salts **1**. As the result of this reaction, two molecules of 1-methyl-3-mercaptoquinoline-4(1*H*)-thione **2** are formed (Scheme 1). This compound was obtained previously via reaction of salt **1** with hydrogen sulfide [27], but its structure has not been confirmed spectroscopically.

**Scheme 2:**
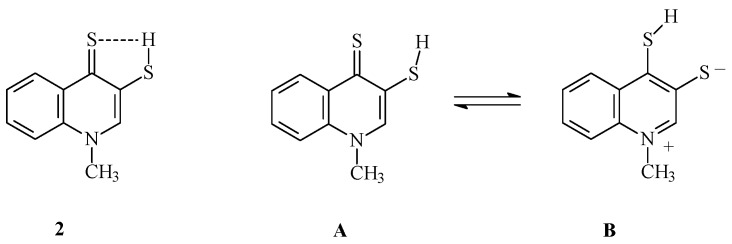
Interaction between thiol proton and thione sulfur atom and the tautomeric structures of compound **2**.

Use of sodium hydrosulfide increases the speed of compound **1** dithiine ring cleavage. This is the result of the stronger nucleophilic properties of hydrosulfide anion as compared to the hydrogen sulfide molecule. The perquisite of reaction with hydrogen sulfide has been the use of pyridine as solvent (proton acceptor). Reaction with NaSH does not require the presence of a proton acceptor (it also proceeds in other solvents, e.g., ethanol, albeit much more slowly). The reaction products are easily soluble in pyridine. ^1^H-NMR spectrum of compound **2** in CDCl_3_ solution lacks a signal from thiol proton, which may be the result of its interaction with thiocarbonyl sulfur atom (C=S) in 4-quinoline position (Scheme 2). This is corroborated by the ^13^C-NMR spectrum of compound **2**, in which C4_quinolinyl_ signal occurs at δ = 134.64 ppm. The signal from thiocarbonyl carbon in ^13^C-NMR spectrum should be present above 180 ppm [28]. It appears that the hydrogen bond does not much affect the IR spectrum of this type of compound. A strong band corresponding to C=S stretching vibrations in compound **2** occurs at 1107 cm^−1^, whereas for compound **3a**, which lacks such interactions, the band is present at 1112 cm^−1^. The elemental composition of compound **2** is confirmed by MS spectrum and the results of elemental analysis.

**Scheme 3:**
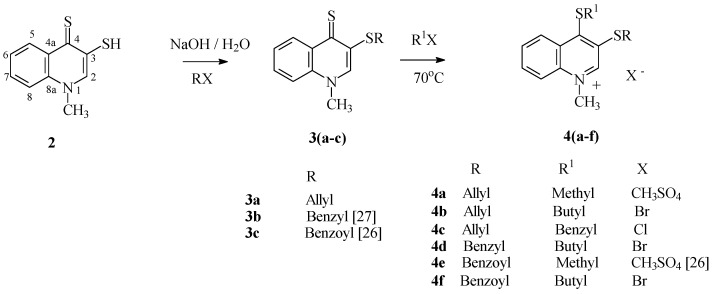
Synthesis of compounds **3** and **4**.

Reactions of **2** in aqueous sodium hydroxide with alkylating or acylating agents (allyl chloride, benzyl chloride and benzoyl chloride) occurred, as expected, on 3-quinoline thiolate sulfur, and led to their respective 1-methyl-3-thioquinoline-4(1*H*)-thione derivatives **3** with 73%–83% yield. The ^13^C-NMR spectra for compounds **3** show signal from the C4_quinolinyl_ carbon occurring in the characteristic range for thiocarbonyl carbon, and this additionally confirms the structure of **2**, the substrate of these reactions. Alkylating of thione function in the 4-quinoline position of compound **3** was carried out at 70 °C, using excess of alkylating agents (dimethyl sulfate, butyl bromide or benzyl chloride) as reaction milieu (Scheme 3). The reactions led to the respective quinoline salts **4** with quantitative yield. 

**Scheme 4:**
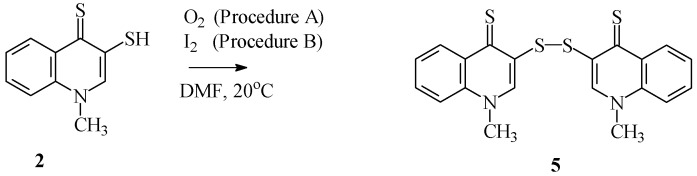
Synthesis of disulfide **5**.

1-Methyl-3-mercaptoquinoline-4(1*H*)-thione **2** in substance is stable, whereas in solution the thiol function is easily oxidized. Compounds **2** were transformed into di(1-methylquinoline-4(1*H*)-thione-3-yl) disulfide **5** by reacting (**2**) in DMF with atmospheric oxygen or ethanolic iodine (Scheme 4). Both procedures gave disulfide **5** with quantitative yield.

**Scheme 5:**
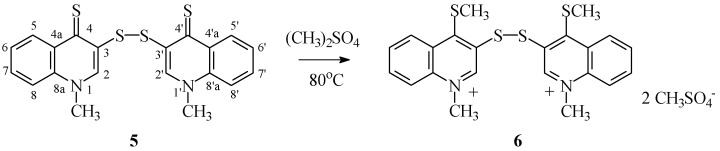
Synthesis of bis-methyl sulfide **6**.

Alkylating disulfide **5** occurred as S-alkylation of both thione groups and led to bis-salts **6** (Scheme 5). The reaction speed is temperature-dependent. At room temperature, only trace reaction is observed. Reaction mixes showed no sign of mono-alkylation products, i.e., resulting from alkylation of a single thione group. As in the case of compounds **3**, the bis-salts **6** were obtained with quantitative yield when reactions were performed at 80 °C and with excess of alkylating agent (dimethyl sulfate) as the reaction milieu.

**Scheme 6:**
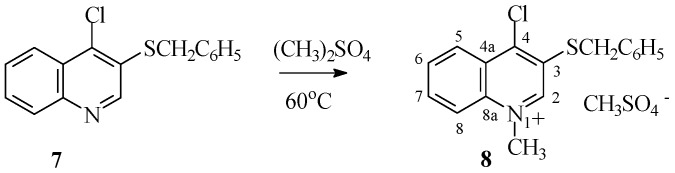
Synthesis of salt **8**.

Salt **8** with chlorine atoms instead of thioalkyl groups in the 4-quinoline position was obtained by *N*-alkylating 4-chloro-3-thiobenzylquinoline **7** derivatives using dimethyl sulfate (Scheme 6). The reaction proceeds easily at 60 °C, leading to salts **8** with quantitative yield.

### 2.2. Biological Activity

The examined derivatives of quinolinium salts demonstrated activity (0.5–512 μg/mL concentration range) against both Gram-positive and Gram-negative bacteria, as well as against *Candida albicans*, a yeast-like fungus. However, greater activity was found against Gram-positive bacteria such as *Staphylococcus*, *Streptococcus*, *Micrococcus* and *Bacillus*. In almost all cases, the tested compounds were more active against *Staphylococcus aureus* methicillin-sensitive strains, although activity also remained very high for methicillin-resistant strains (MRSA). Most likely, the high activity of the tested compounds results from structural features of the bacterial wall. In the case of Gram-negative bacteria, the examined compounds were active in the 8–512 μg/mL range. As in the case of Gram-positive bacteria, the highest activity against Gram-negative strains was demonstrated by derivatives **4d** and **4f**. The lowest activity of sulfur derivatives of quinolinium salts was found for Gram-negative bacteria of *Pseudomonas* genus. Compounds **4d** and **4f** were also highly active against yeast-like fungus *Candida albicans* (MIC = 1). In order to examine the effect of thioalkyl group on the activity of the tested compounds their structure was modified via introduction of chlorine atoms instead of thioalkyl groups into the 4-quinoline position (compound **6**), as well as introducing disulfide groups in the 3-quinoline position (compound **8**), respectively. The obtained results demonstrate that the lack of the thioalkyl group in the 4-quinoline position considerably decreases antimicrobial activity, whereas the compound with the disulfide group shows activity comparable to those of compounds **4**(**a**–**c**), i.e., derivatives containing the allylthio group in the 3-position.

We previously reported the activity of 1,3,4-trisubstituted 4-aminoquinoline salts [22,23]. These compounds also demonstrated activity against Gram-positive and Gram-negative bacteria. However, the structural modification achieved by introducing thioalkyl substituents instead of alkylamine ones in the 4-quinoline position markedly increased their antibacterial and antifungal activity. The MIC values reported here for Gram-positive bacteria are comparable to those of ciprofloxacin (reference). This may suggest the need for a continuing search effort for novel types of sulfur derivatives of quinoline salts, as well as the assessment of antibacterial activity using not only standard strains, but also clinically relevant ones.

## 3. Materials and Methods

### 3.1. Chemistry

Melting points are uncorrected. NMR spectra were recorded using a Bruker Ascend 600 spectrometer (Bruker, Billerica, MA, USA). To assign the structures, the following 2D experiments were employed: ^1^H-^13^C gradient selected HSQC and HMBC sequences. Standard experimental conditions and standard Bruker programs were used. The ^1^H- and ^13^C-NMR spectral data are given relative to the TMS signal at 0.0 ppm. HR mass spectra were recorded with Bruker Impact II (Bruker, Billerica, MA, USA). Solid-state infrared spectra were recorded in the range of 4000–1000 cm^−1^ using the Shimadzu IRAffinity-1 FTIR spectrometer (Shimadzu, Kyoto, Japan) and KBr pellet method. CHNS elemental analysis was performed using EuroVector 3018 analyser (EuroVector, Pavia, Italy). Total halides were determined using titration (after mineralization) with mercuric nitrate solution. 

#### 3.1.1. Synthesis of 1-Methyl-3-(mercapto)quinoline-4(1*H*)-thione **2**

Sodium hydrosulfide hydrate (2.5 mmol) was added to the mixture of bis-chloride (**1**) (0.419 g, 1 mmol) in 10 mL of dry pyridine, and the whole was mixed at 20 °C for 1 h. The mixture was poured into 100 mL of water. The formed precipitate was filtered off, washed with water and dried over calcium chloride in desiccator with concomitant argon atmosphere. 

*1-Methyl-3-(mercapto)quinoline-4(1H)-thione* (**2**). Yield 87%; m.p. 258 °C; IR (KBr, cm^−1^): ν_max_: 1107 (C=S); (CDCl_3_), 600 MHz) δ (ppm): 4.01 (s, 3H, NCH_3_), 7.50–7.54 (m, 1H, H6), 7.54–7.58 (m, 1H, H8), 7.68–7.72 (m, 1H, H7), 8.06 (s, 1H, H2), 8.97–9.01 (m, 1H, H5); ^13^C-NMR (DMSO_d-6_, 150.9 MHz) δ (ppm): 42.30 (NCH_3_), 116.16 (C8), 126.37 (C6), 126.89 (C3), 130.82 (8a), 131.36 (C7), 131.64 (4a), 132.37 (C5), 133.20 (C2), 134.64 (C4); ESI-HRMS Calcd for C_10_H_10_NS_2_ ([M + H]^+^): 208.0254, Found: 208.0254; Anal. Calcd for C_10_H_9_NS_2_: C 57.94, H 4.38, N 6.76, S 30.93. Found: C 57.87, H 4.31, N 6.73, S 30.85.

#### 3.1.2. General Procedure for Synthesis of 1-Methyl-3-thioquinoline-4(1*H*)-thione Derivatives **3**

1 mmol (0.207 g) 1-methyl-3-mercaptoquinoline-4(1*H*)-thione (**2**) was added dropwise with stirring into 50 mL of 5% aqueous sodium hydroxide. An alkylating agent or acylating (1.25 mmol) was added dropwise with stirring for 1 h. The formed precipitate was filtered off, washed with water and dried over calcium chloride in vacuum desiccator. The raw product was purified through recrystallization from ethanol.

*1-Methyl-3-(allylthio)quinoline-4(1H)-thione* (**3a**). Yield 78%; m.p. 212–214 °C; ^1^H-NMR (DMSO_d-6_, 600 MHz) δ (ppm): 3.66 (d*, J* = 6.6 Hz, 2H, SCH_2_CH), 4.08 (s, 3H, NCH_3_), 5.10-5-18 (m, 1H, CH=CH_2_), 5.23–5.35 (m, 1H, CH=CH_2_), 5,78–6.00 (m, 1H, CH=CH_2_), 7.48–7.58 (m, 1H, H6), 7.75–7.82 (m, H, H7), 7.83–7.88 (m, 1H, H8), 8.02 (s, 1H, H2), 8.76–8.83 (m, 1H, H5); ^13^C-NMR (DMSO_d-6_, 150.9 MHz) δ: 34.46 (SCH_2_), 42.17 (NCH_3_), 118.23 (C8), 118.62 (CH_2_=CH), 126.17 (C6), 129.31 (C5), 131.54 (C4a), 132.07 (CH_2_=CH), 132.11 (C7), 134.05 (C3), 135.31 (C8a) 136.12 (C2), 187.09 (C4); ESI-HRMS Calcd for C_13_H_14_NS_2_ ([M + H]^+^): 248.0567, Found: 248.0564; Anal. Calcd for C_13_H_13_NS_2_: C 63.12, H 5.30, N 5.66, S 25.92. Found: C 63.07, H 5.24, N 5.58, S 25.89.

*1-Methyl-3-(benzylthio)quinoline-4(1H)-thione* (**3b**). Yield 83%; m.p. 204–206 °C; ^1^H-NMR (DMSO_d-6_, 600 MHz) δ (ppm): 4.07 (s, 3H, NCH_3_), 4.21 (s, 2H, SCH_2_), 7.32–7.37 (m, 3H, H_arom_), 7.41–7.44 (m, 2H, H_arom_), 7.53–7.58 (m, 1H, H6), 7.79–7.83 (m, 1H, H7), 7.85–7.89 (m, 1H, H8), 8.12 (s, 1H, H2), 8.79–8.83 (m, 1H, H5); ^13^C-NMR (DMSO_d-6_, 150.9 MHz) δ (ppm): 36.23 (CH_2_), 42.18 (NCH_3_), 118.28 (C8), 126.21 (C6), 127.60 (H1_benzyl_), 128.98 (C2,C6_benzyl_), 129.32 (C5), 129.60 (C3,C5_benzyl_), 132.03 (C4_benzyl_), 132.132.15 (C7), 132.46 (C4a), 135.34 (C8a), (C2), 137.50 (C3), 186.65 (C4); ESI-HRMS Calcd for C_17_H_15_NS_2_ ([M + H]^+^): 298.0724, Found: 298.0714; Anal. Calcd for C_17_H_15_NS_2_: C 68.65, H 5.08, N 4.71, S 21.56. Found: C 68.54, H 5.01, N 4.65, S 21.50.

*1-Methyl-3-(benzoylthio)quinoline-4(1H)-thione* (**3c**). Yield 73%; m.p. 210–212 °C; ^1^H-NMR (DMSO_d-6_, 600 MHz) δ (ppm): 4.07 (s, 3H, NCH_3_), 7.60–7.67 (m, 3H, H_arom_), 7.72–7.78 (m, 1H, H_arom_), 7.88–7.95 (m, 2H, H_arom_), 8.01–8.05 (m, 1H, H8), 8.68 (s, 1H, H2), 8.86–8.90 (m, 1H, H5); ^13^C-NMR (DMSO_d-6_, 150.9 MHz) δ (ppm): 41.94 (NCH_3_), 118.68 (C8), 121.41 (C1_benzoyl_), 127.07 (C4_benzoyl_), 127.56 (C3,C5_benzoyl_), 129.74 (C2,C6_benzoyl_), 130.73 (C5), 133.33 (C7), 133.45 (C3), 134.60 (C6), 136.39 (C4a), 136.66 (C8a), 145.68 (C2), 188.99 (CO), 193.19 (C4); ESI-HRMS Calcd for C_17_H_14_NOS_2_ ([M + H]^+^): 312.0517, Found: 312.0515; Anal. Calcd for C_17_H_13_NOS_2_: C 65.57, H 4.21, N 4.50, S 20.59. Found: C 65.51, H 4.14, N 4.47, S 20.55.

#### 3.1.3. General Procedure for Synthesis of 1-Methyl-3-thio-4-thioquinolinium Derivatives Salts (**4**)

The mixture (suspension) of thione (**3**) (1 mmol) and alkylating agents (5 mmol) was stirred and heated at 80 °C for 3 h. Unreacted alkylating agents were removed by vacuum distillation. Then 5 mL of dry ethanol was added. The mixture was refluxed for 5 min and cooled down to room temperature. The solid was filtered off and washed with dry ethanol.

*1-Methyl-3-(allylthio)-4-(methylthio)quinolinium methyl sulfate* (**4a**). Yield 95%; oil; ^1^H-NMR (D_2_O, 600 MHz) δ (ppm): 2.53 (s, 3H, SCH_3_), 3.55 (s, 3H, OCH_3_), 3.67–3.74 (d, 2H, *J* = 6.6 Hz, SCH_2_CH), 4.41 (s, 3H, NCH_3_), 5.00–5.05 (m, 1H, CH=CH_2_), 5.09–5.17 (m 1H, CH=CH_2_), 5.74–5.85 (m, 1H, CH=CH_2_), 7.75–7.79 (m, 1H, H6), 7.93–7.98 (m, 1H, H7), 8.08–8.13 (m, 1H, H8), 8.44–8.48 (m, 1H, H5), 8.78 (s, 1H, H2); ^13^C-NMR (D_2_O, 150.9 MHz) δ (ppm): 19.38 (OCH_3_), 35.95 (SCH_2_), 45.07 (NCH_3_), 55.31 (SCH_3_), 118.81 (C8), 119.36 (CH=CH_2_),127.56 (C5), 130.03 (C4a), 130.41 (C6), 131.89 (CH=CH_2_), 134.71 (C7), 135.05 (C8a), 136.27 (C3), 146.48 (C2), 158.91 (C4); ESI-HRMS Calcd for C_14_H_16_NS_2_ ([M]^+^): 262.0724, Found: 262.0722; Anal. Calcd for C_15_H_19_NO_4_S_3_: C 48.24, H 5.13, N 3.75, S 25.75. Found: C 48.20, H 5.08, N 3.67, S 25.70.

*1-Methyl-3-allylthio-4-(butylthio)quinolinium bromide* (**4b**). Yield 91%; m.p. 168 °C dec.; ^1^H-NMR (D_2_O, 600 MHz) δ (ppm): 0.55–0.750 (t, *J* = 7.2 Hz, 3H, CH_2_CH_3_), 1.10–1.25 (m, 2H, CH_2_CH_3_), 1.25–1.40 (m, 2H, SCH_2_CH_2_), 3.00–3.12 (t, *J* = 7.2 Hz, SCH_2_CH_2_), 3.70–3.78 (d, 2H, *J* = 6.0 Hz, SCH_2_CH), 4.43 (s, 3H, NCH_3_), 5.03–5.08 (m, 1H, CH=CH_2_), 5.12–5.20 (m 1H, CH=CH_2_), 5.73–5.84 (m, 1H, CH=CH_2_), 7.73–7.82 (m, 1H, H6), 7.91–8.00 (m, 1H, H7), 8.08–8.12 (m, 1H, H8), 8.48–8.54 (m, 1H, H5), 8.79 (s, 1H, H2); ^13^C-NMR (D_2_O, 150.9 MHz) δ (ppm): 12.63 (CH_3_CH_2_), 21.03 (CH_3_CH_2_), 31.54 (SCH_2_CH_2_), 35.72 (SCH_2_CH), 37.06 (SCH_2_CH_2_), 45.22 (NCH_3_), 118.80 (C8), 119.45 (CH=CH_2_), 127.72 (C5), 130.53 (C6), 130.86 (C4a), 131.80 (CH=CH_2_), 134.65 (C7), 136.15 (C8a), 136.55 (C3), 145.84 (C2), 156.69 (C4); ESI-HRMS Calcd for C_17_H_22_NS_2_ ([M]^+^): 304.1193, Found: 304.1191; Anal. Calcd for C_17_H_22_BrNS_2_: C 53.12, H 5.77, Br 20.79, N 3.64, S 16.68. Found: C 53.05, H 5.71, Br 20.70, N 3.59, S 16.63.

*1-Methyl-3-allylthio-4-(benzylthio)quinolinium chloride* (**4c**). Yield 93%; m.p 183 °C dec; ^1^H-NMR (D_2_O, 600 MHz) δ (ppm): 3.62–3.68 (d, *J* = 6.6 Hz, 2H, SCH_2_CH), 4.02 (s, 2H, SCH_2_), 4.32 (s, 3H, NCH_3_), 5.02–5.09 (m, 1H, CH=CH_2_), 5.11–5.18 (m, 1H, CH=CH_2_), 5.64–5.75 (m, 1H CH=CH_2_), 6.71–6.76 (m, 2H, H_benzyl_), 6.81–6.90 (m, 3H, H_benzyl_), 7.58–7.64 (m, 1H, H6), 7.83–7.89 (m, 1H, H7), 7.96–8.01 (m, 1H, H8), 8.13–8.18 (m, 1H, H5), 8.65 (s, 1H, H2); ^13^C-NMR (D_2_O, 150.9 MHz) δ (ppm): 35.32 (SCH_2_CH), 40.23 (SCH_2_), 45.44 (NCH_3_), 118.73 (C8), 119.83 (CH=CH_2_), 127.42 (C4_benzyl_), 127.71 (C5), 128.41 (C2,C6_benzyl_), 128.76 (C3,C5_benzyl_), 128.86 (C1_benzyl_), 130.72 (C6), 131.09 (C4a), 131.47 (CH=CH_2_), 134.60 (C7), 135.79 (C8a), 138.25 (C3), 145.13 (C2), 152.90 (C4); ESI-HRMS Calcd for C_20_H_20_NS_2_ ([M]^+^): 338.1037, Found: 338.1037; Anal. Calcd for C_20_H_20_ClNS_2_: C 64.24, H 5.39, Cl 9.48, N 3.75, S 17.15. Found: C 64.17, H 5.35, Cl 9.41, N 3.69, S 17.11.

*1-Methyl-3-benzylthio-4-(butylthio)quinolinium bromide* (**4d**). Yield 90%; m.p. 140 °C dec.; ^1^H-NMR (DMSO_d-6_, 600 MHz) δ(ppm): 0.73–0.82 (t, *J* = 7.2 Hz, 3H, CH_2_CH_3_), 1.28–1.37 (m, 2H, CH_2_CH_3_), 1.39–1.46 (m, 2H, SCH_2_CH_2_), 3.15–3.20 (t, *J* = 7.2 Hz, 2H, SCH_2_CH_2_), 4.64 (s, 1H, SCH_2_), 4.67 (s, 3H, NCH_3_), 7.28–7.34 (m, 1H, H4_benzyl_), 7.34–7.40 (m, 2H, H3,H5_benzyl_), 7.45–7.48 (m, 2H, H2,H6_benzyl_), 8.05–8.10 (m, 1H, H6), 8.20–8.23 (m, 1H, H7), 8.47–8.51 (m, 1H, H8), 8.70–8.74 (m, 1H, H5), 9.56 (s, 1H, H2); ^13^C-NMR (DMSO_d-6_, 150.9 MHz) δ (ppm): 13.76 (CH_3_CH_2_), 21.56 (CH_3_CH_2_), 32.10 (SCH_2_CH_2_), 36.80 (SCH_2_), 37.43 (SCH_2_CH_2_) 45.81 (NCH_3_), 120.53 (C8), 127.68 (C5), 128.34 (C4_benzyl_), 129.32 (C3,C5_benzyl_), 129.68 (C2,C6_benzyl_), 130.92 (C4a), 131.35 (C6), 134.59 (C1_benzyl_), 135.93 (C7), 136.41 (C8a), 138.36 (C3), 147.06 (C2), 153.07 (C4); ESI-HRMS Calcd for C_21_H_24_NS_2_ ([M]^+^): 354.1350, Found: 354.1351; Anal. Calcd for C_21_H_24_BrNS_2_: C 58.06, H 5.57, Br 18.39, N 3.22, S 14.76. Found: C 57.91, H 5.50, Br 18.35, N 3.15, S 14.74.

*1-Methyl-3-benzoylthio-4-(methylthio)quinolinium methyl sulfate* (**4e**). Yield 95%; m.p. 98 °C dec.; ^1^H-NMR (D_2_O, 600 MHz) δ (ppm): 2.53 (s, 3H, SCH_3_), 3.51 (s, 3H, OCH_3_), 4.36 (s, 3H, NCH_3_), 7.25–7.31 (m, 2H, H3,H5_benzoyl_), 7.44–7.49 (m, 1H, H4_benzoyl_), 7.70–7.78 (m, 2H, H2,H6_benzoyl_), 7.80–7.86 (m, 1H, H6), 8.04–8.09 (m, 1H, H7), 8.12–8.17 (m, 1H, H8), 8.47–8.52 (m, 1H, H5), 9.03 (s, 1H, H2); ^13^C-NMR (D_2_O, 150.9 MHz) δ (ppm): 20.88 (SCH_3_), 44.93 NCH_3_), 55.28 (OCH_3_), 119.13 (C8), 125.11 (C3), 127.58 (C3,C5_benzoyl_), 128.60 (C5), 129.14 (C2,C6_benzoyl_), 129.66 (C4a), 130.63 (C6), 134.29 (C1_benzoyl_), 135.30 (C4_benzoyl_), 136.50 (C7), 137.39 (C8a), 151.46 (C2), 167.62 (C4), 189.08 (CO), ESI-HRMS Calcd for C_18_H_16_NOS_2_ ([M]^+^): 326.0673, Found: 326.0672; Anal. Calcd for C_19_H_19_NO_5_S_3_: C 52.16, H 4.38, N 3.20, S 21.98: Found: 52.08, H 4.31, N 3.15, S 21.95.

*1-Methyl-3-benzoylthio-4-(butylthio)quinolinium bromide* (**4f**). Yield 89%; m.p. 112 °C dec.; ^1^H-NMR (DMSO_d-6_, 600 MHz) δ (ppm): 0.45–0.53 (t, *J* = 7.2 Hz, 3H, CH_2_CH_3_), 1.00–1.11 (m, 2H, CH_2_CH_3_), 1.19–1.25 (m, 2H, SCH_2_CH_2_), 3.00–3.09 (t, *J* = 7.2 Hz, 2H, SCH_2_CH_2_), 4.43 (s, 3H, NCH_3_), 7.35–7.40 (m, 2H, H3,H5_benzoyl_), 7.53–7.57 (m, 1H, H4_benzoyl_), 7.80–7.84 (m, 2H, H2,H6_benzoyl_),. 7.84–7.88 (m, 1H, H6), 8.07–8.12 (m, 1H, H7), 8.18–8.23 (m, 1H, H8), 8.60–8.64 (m, 1H, H5), 9.14 (s, 1H, H2); ^13^C-NMR (DMSO_d-6_, 150.9 MHz) δ (ppm): 12.52 (CH_2_CH_3_), 20.91 (CH_2_CH_3_), 31.54 (SCH_2_CH_2_), 38.79 (SCH_2_CH_2_), 45.08 (NCH_3_), 119.16 (C8), 126.69 (C3), 127.64 (C3,C5_benzoyl_), 129.00 (C5), 129.24 (C2,C6_benzoyl_), 130.57 (C4a), 130.77 (C6), 134.42 (C1_benzoyl_), 135.39 (C4_benzoyl_), 136.53 (C7), 137.56 (C8a), 151.63 (C2), 166.03 (C4), 189.51 (CO); ESI-HRMS Calcd for C_21_H_22_NOS_2_ ([M]^+^): 368.1143, Found: 368.1134; Anal. Calcd for C_21_H_22_BrNOS_2_: C 56.25, H 4.94, Br 17.82, N 3.12, S 14.30. Found: C 56.14, H 4.86, Br 17.74, N 3.07, S 14.25.

#### 3.1.4. Synthesis of Di(1-methylquinoline-4(1*H*)-thione-3-yl) Disulfide (**5**)

Procedure (A). Air was passed through the solution of 1-methyl-3-mercaptoquinoline-4(1*H*)-thione (**2**) (1 mmol, 0.207g) in dry DMF (50 mL) at rt over 1 h. The solid product was filtered off and washed with dry ether. The mixture of raw product and 10 mL dry ethanol was refluxed for 5 min and cooled down to room temperature. The solid product was filtered off and washed with dry ethanol.

Procedure (B). To the solution of 1-methyl-3-mercaptoquinoline-4(1*H*)-thione (**2**) (1 mmol, 0.207 g) in dry DMF (50 mL) a solution of 1.5 mmol (0.381 g) of iodine in 5 mL of ethanol was added over 1 h with stirring (rt). The solid product was filtered off and washed with dry ether. The mixture of raw product and 10 mL dry ethanol was refluxed for 5 min and cooled down to room temperature. The solid product was filtered off and washed with dry ethanol.

*Di(1-methylquinoline-4(1H)-thione-3-yl) disulfide* (**5**). Yield: Procedure A: 92%, Procedure B: 95%; m.p. 301–302 °C; ^1^H-NMR (DMSO_d-6_, 600 MHz) δ (ppm): 4.05 (s, 6H, NCH_3_, N′CH_3_), 7.60–7.65 (m, 2H, H6, H6′), 7.83–7.87 (m, 2H, H7, H7′), 7.90–7.94 (m, 2H, H8, H8′), 8.34 (s, 2H, H2,H2′), 8.76–8.80 (m, 2H, H5,H5′); ESI-HRMS Calcd for C_20_H_16_N_2_S_4_ ([M]^2+^): 206.0098, Found: 206.0092; Anal. Calcd for C_20_H_16_N_2_S_4_: C 58.22, H 3.91, N 6.79, S 31.08. Found: C 58.17, H 3.86, N 6.74, S 31.05.

#### 3.1.5. Synthesis of Bis-Methyl Sulfate di(1-methyl-4-(methylthio)quinolinium-3-yl) Disulfide **6**

The mixture of compounds (**5**) (1 mmol) and dimethyl sulfate (5 mmol) was stirred and heated at 80 °C for 2 h. Then, 10 mL of dry ethanol was added. The mixture was refluxed for 5 min and cooled down to room temperature. The solid was filtered off and washed with dry ethanol. The raw product was purified through recrystallization from ethanol.

*Bis-methyl sulfate di(1-methyl-4-(methylthio)quinolinium-3-yl) disulfide* (**6**) Yield 94%; m.p. 120 °C dec.; ^1^H-NMR (DMSO_d-6_, 600 MHz) δ (ppm): 2.80 (s, 6H, OCH_3_), 3.31 (s, 6H, SCH_3_,S′CH_3_), 4.62 (s, 6H, NCH_3_,N′CH_3_), 8.12–8.15 (m, 2H, H6,H′6), 8.29–8.33 (m, 2H, H7,H′7), 8.53–8.56 (m, 2H, H8,H′8), 8.72–8.76 (m, 2H, H5,H′5), 9.62 (s, 2H, H2,H′2);.^13^C-NMR (DMSO_d-6_, 150.9 MHz) δ (ppm): 21.24 (C), 46.14 (C), 53.24 (C), 120.77 (C8), 128.15 (C5), 130.02 (C4a), 131.58 (C6), 135.14 (C7), 135.89 (C3), 137.65 (C8a), 148.46 (C2), 158.13 (C4); ESI-HRMS Calcd for C_22_H_22_N_2_S_4_ ([M]^2+^): 221.0333, Found: 221.0322; Anal. Calcd for C_24_H_28_N_2_O_8_S_6_: C 43.36, H 4.24, N 4.21, S 28.93, Found: C 43.27, H 4.19, N 4.17, S 28.90.

#### 3.1.6. Synthesis of 1-Methyl-4-chloro-3-(benzylthio)quinolinium Methyl Sulfate (**8**)

The mixture (suspension) of compounds (**7**) (1 mmol) and dimethyl sulfate (5 mmol) was stirred and heated at 80 °C for 2 h. Then, 5mL of dry ethanol was added. The mixture was refluxed for 5 min and cooled down to the room temperature. The solid was filtered off and washed with dry ethanol. The raw product was purified through recrystallization from ethanol.

*1-Methyl-4-chloro-3-(benzylthio)quinolinium methyl sulfate* (**8**). Yield 89%; m.p. 204 °C dec.; ^1^H-NMR (DMSO_d-6_, 600 MHz) δ (ppm): 3.70 (s, 3H, OCH_3_.), 4.64 (s,2H, CH_2_), 4.67 (s, 3H, NCH_3_), 7.26–7.32 (m, 3H, H4_benzyl_), 7.32–7.43 (m, 2H, H3,H5_benzyl_), 7.43–7.50 (m, 2H, H2,H6_benzyl_) 8.10–8.18 (m, 1H, H6), 8.23–8.27 (m, 1H, H7), 8.48–8.59 (m, 2H, H5,H8), 9.68 (s, 1H, H2), ^13^C-NMR (DMSO_d-6_, 150.9 MHz) δ ppm): 37.32 (CH_2_), 45.95 (NCH_3_), 53.29 (OCH_3_), 120.59 (C5), 126.02 (C8), 127.22 (C4a), 128.43 (C4_benzyl_), 129.38 (C3,C5_benzyl_), 129.70 (C2,C6_benzyl_), 132.21 (C6), 132.29 (C1_benzyl_), 135.54 (C7), 135.63 (C3), 137.46 (C8a), 148.67 (C4), 149.46 (C2); ESI-HRMS Calcd for C_18_H_18_ClNS ([M]^+^): 300.0614, Found: 300.0612; Anal. Calcd for C_18_H_18_ClNO_4_S_2_: C 52.49, H 4.40, Cl 8.61, N 3.40, S 15.57. Found: C 52.38, H 4.36, Cl 8.54, N 3.37, S 15.51.

### 3.2. Biological Assays

Analysis of antimicrobial activity was performed using the serial microdilution method and Muelller-Hinton broth (MHB). Thirteen standard strains from ATTC collection were used (*Staphylococcus aureus* ATCC 25923 (SA25923), *Staphylococcus aureus* ATCC 43300 (SA43300), *Staphylococccus epidermidis* ATCC 12228 (SE12228), *Enterococcus faecalis* ATCC 29212 (E29212), *Micrococcus luteus* ATCC 10240 (ML10240), *Bacillus subtilis* ATCC 11774 (BS11774), *Escherichia coli* ATCC 11776 (EC11776), *Klebsiella pneumoniae* ATCC 27736 (KP27736), *Proteus mirabilis* ATCC 7002 (PM7002), *Pseudomonas aeruginosa* ATCC 27853 (PA27853), *Acinetobacter baumannii* ATCC 19606 (AB19606), *Serratia marcescens* ATCC 8100 (SM8100) and *Candida albicans* ATCC 60193 (CA60193). An inoculum density of 5 × 10^5^ cfu/mL was used. Antimicrobial activity was examined using 96-well titration plates. The concentration range of the examined sulfur derivatives of quinolinium salts was 0.5–512 μg/mL As control, ciprofloxacin (CIP) was used (0.001–2 μg/mL range). MIC determination was performed for 20-h incubation periods at 37 °C. Tests were performed in accordance with EUCAST recommendations [29]. The results are presented in Table 1.

## 4. Conclusions

Reaction of thioquinanthrenium salts **1** with sodium hydrosulfide leads to cleavage of the dithiin ring and the formation of 1-methyl-3-mercaptoquinoline-4(1*H*)-thione **2**. Subsequent reactions of the thiol and thione groups in compound **2** allow the series of sulfide and disulfide derivatives of quinolinium salts **4** and **6** to be obtained. 1-Methyl-4-chloro-3-(benzyllthio)quinoline salt **8** can be synthesized by *N*-alkylating of 4-chloro-3-(benzylthio)quinoline using dimethyl sulfate. The obtained compounds demonstrated greater activity against Gram-negative bacteria than Gram-positive ones. Introduction of sulfur substituents into 4-quinoline position markedly increased activity compared to previously reported 4-aminoquinoline derivatives. Compounds **4d** and **4f** with thiobenzyl and thiobenzoyl substituents in the 3-quinoline position demonstrated activity comparable to that of ciprofloxacine, used as reference. 

The MIC values exhibited by these compounds warrant the continuing search for novel 1,3,4-trisubstituted quinoline salts with antimicrobial activity.

## Figures and Tables

**Table 1:** Evaluation of the minimum inhibitory concentration (MIC) (μg/mL) of quinolinium salt sulfur derivatives against tested bacterial strains.

	4a	4b	4c	4d	4e	4f	6	8	CIP
*S. aureus* ATCC 25923	32	32	16	0.5	1	1	16	64	0.25
*S. aureus* ATCC 43300	128	128	16	1	32	4	32	32	0.5
*S. epidermidis* ATCC 12228	32	128	32	0.5	2	1	32	128	0.25
*E. faecalis* ATCC 29212	128	128	32	0.5	1	1	32	32	0.5
*M. luteus* ATCC 10240	64	128	32	0.5	1	0.5	64	16	1
*B. subtilis* ATCC 11774	64	128	32	0.5	4	0.5	16	256	2
*E. coli* ATCC 11776	64	128	128	16	64	32	32	128	0.016
*K. pneumoniae* ATCC 27736	64	128	128	32	128	64	256	256	0.5
*P. mirabilis* ATCC 7002	128	128	128	8	128	32	128	128	0.25
*P. aeruginosa* ATCC 27853	256	256	128	64	512	256	256	256	0.5
*A. baumannii* ATCC 19606	128	128	64	8	16	8	16	256	0.25
*S. marcescens* ATCC 8100	64	64	128	16	64	8	64	128	1
*C. albicans* ATCC 60193	64	64	64	1	16	1	16	128	nt

nt-not tested.
